# Granulomatosis With Polyangiitis: A Rare Cause of Recurrent Breast Abscesses

**DOI:** 10.7759/cureus.64993

**Published:** 2024-07-20

**Authors:** Rishi Bothara, Imran Umer, Alisha Bhalodia, Muneeb Rehman, Zain Amar

**Affiliations:** 1 Internal Medicine, University of Illinois, Peoria, USA; 2 Rheumatology, University of Illinois, Peoria, USA; 3 Internal Medicine, Isra University, Hyderabad, PAK

**Keywords:** non-caseating granuloma, scleritis, methotrexate, breast abscess, granulomatosis with polyangiitis (gpa)

## Abstract

This case report highlights the importance of recognizing granulomatosis with polyangiitis (GPA) as a rare but potential cause of recurrent granulomatous mastitis and breast abscesses. The case report describes a patient who presented with recurrent breast abscesses over many years, initially misdiagnosed as infectious mastitis, until a breast tissue biopsy revealed granulomatous inflammation. Further evaluation confirmed the diagnosis of GPA based on elevated anti-neutrophil cytoplasmic antibodies (ANCA). The authors emphasize that, while breast involvement is exceedingly rare in GPA, it should be considered in cases of refractory granulomatous mastitis, even in the absence of typical systemic GPA manifestations. Early recognition can prevent misdiagnosis, allow timely initiation of immunosuppressive treatment, and avoid unnecessary procedures. The report calls for improved awareness and further research into the clinical characteristics and optimal management strategies for GPA presenting with breast lesions.

## Introduction

Granulomatosis with polyangiitis (GPA), previously known as Wegener's granulomatosis, is a multisystem vasculitis that can affect different organ systems. It most commonly affects the upper and lower respiratory tract, kidneys, and ear, nose, and throat (ENT) region, causing manifestations such as saddle nose deformity, rhinosinusitis, septal perforation, subglottic stenosis, pulmonary nodules, and diffuse alveolar hemorrhage. Histopathologically, it presents as focal granulomatous necrotizing vasculitis affecting small- to medium-sized vessels and is often associated with antineutrophil cytoplasmic antibodies directed against serine proteinase 3 (PR3) [1,2]. Involvement of the breast in GPA is considered very uncommon, with only a few documented case reports in the medical literature. This case report presents a patient with recurrent breast abscess, which on breast tissue biopsy turned out to be granulomatous mastitis, eventually diagnosed as GPA-related granulomatous mastitis.

## Case presentation

A 37-year-old woman presented to the clinic with a history of recurrent breast abscesses requiring incision and drainage. According to the patient, she experienced her first episode of left breast pain and swelling at the age of 26 during her initial pregnancy. This incident was successfully treated with ibuprofen, and the symptoms resolved.

The patient remained asymptomatic until her second pregnancy at the age of 33, during which she encountered a recurrent breast mass with tenderness. This episode was managed with heat packs and Tylenol. Subsequently, two years later, she faced a resurgence of symptoms, leading her to seek medical attention from her primary care physician. The diagnosis at that point was mastitis with a breast abscess. She underwent incision and drainage of the abscess and received a course of oral antibiotics, which completely resolved her symptoms.

**Figure 1:**
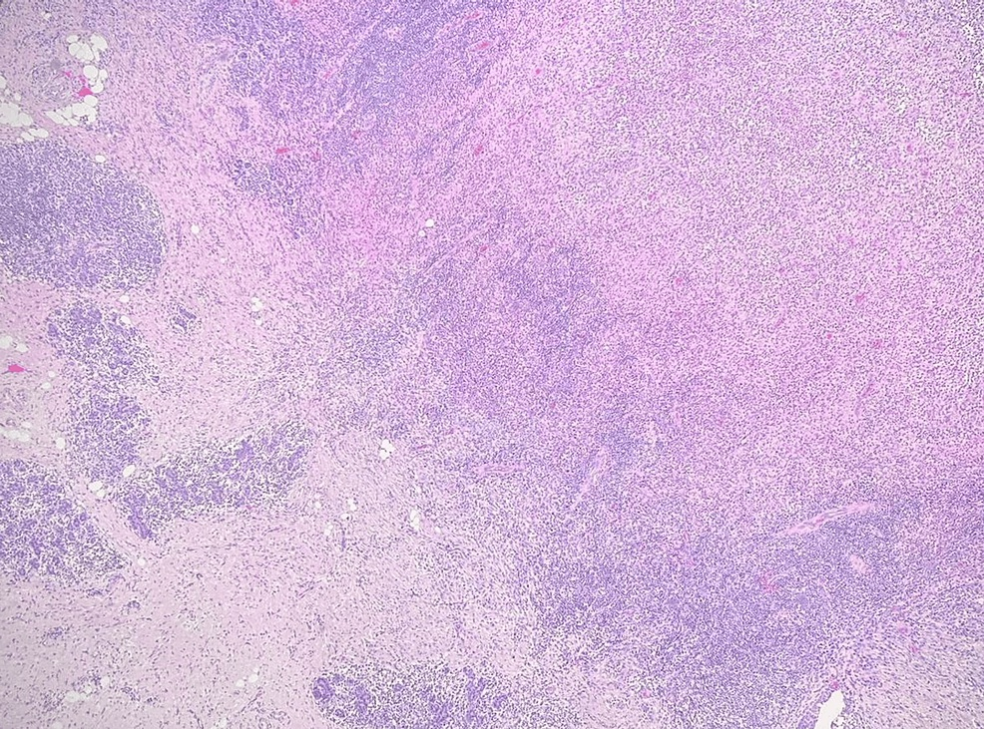
Underlying breast tissue with florid perilobular mixed inflammatory infiltrates consisting of lymphocytes, histiocytes, and neutrophils

**Figure 2:**
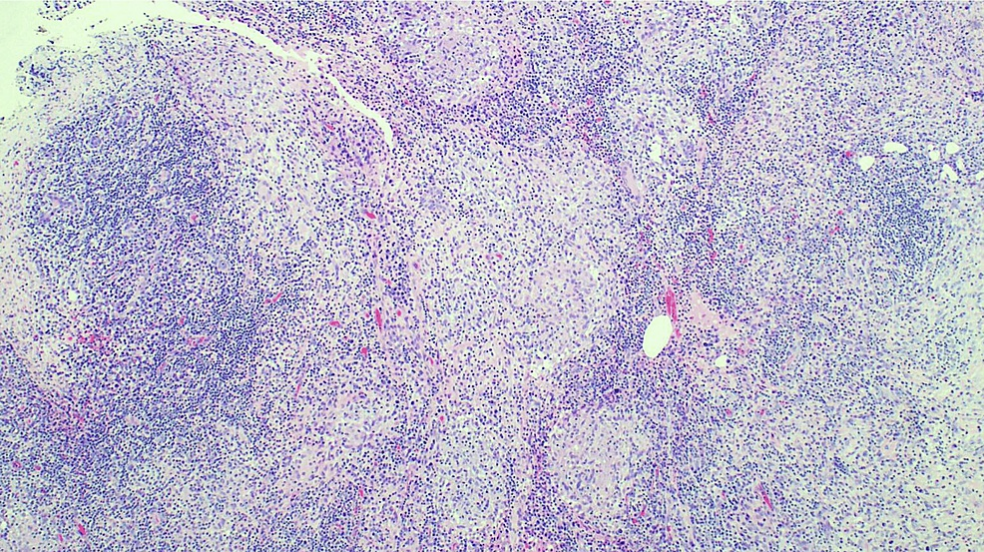
Multiple non-caseating granulomas comprised of epithelioid histiocytes and surrounded by lymphocytes

Over the following year, she continued to experience recurrent symptoms of breast infection with abscesses, resulting in multiple incision and drainage procedures and antibiotic courses. During this period, an ultrasound-guided biopsy of the breast mass was conducted, revealing benign breast tissue with mixed inflammatory infiltrates, comprised of histiocytes with admixed lymphocytes, neutrophils, eosinophils, and plasma cells, as seen in Figure 1. Multiple non-caseating granulomas consist of epithelioid histiocytes and lymphocytes (Figure 2). Gram stain and tissue culture were negative for bacterial growth. Periodic acid-Schiff with diastase (PAS-D) and Grocott methenamine silver-nitrate (GMS) stains were negative for fungal organisms, and acid-fast bacilli (AFB) stains were negative for acid-fast bacilli. The diagnosis of granulomatous mastitis was established and prompted a referral to rheumatology for an autoimmune workup.

During the evaluation, the patient denied experiencing similar issues in her axilla, groin, or other areas. She has had episodes of mild sinus infections in the remote past but no history of other upper airway or pneumonia. She also denied accompanying symptoms such as skin rash, photosensitivity, joint pain, oral sores, nasal ulcers, dry skin, dry mouth, muscle aches, chest pain, or abdominal pain. A diagnostic workup was done. The patient’s blood count and metabolic profile were unremarkable. Erythrocyte sedimentation rate (ESR) and C-reactive protein (CRP) were within normal range. All other autoimmune workups were negative, except for the cytoplasmic antineutrophil cytoplasmic antibody (c-ANCA), which was elevated at a titer of 1:80 with the PR3 antibody. Other extensive workups were unremarkable for serum prolactin, fungal infections, sarcoidosis, and tuberculosis.

The patient was initiated on oral prednisone 20 mg daily, and during the follow-up visit at the end of two months, she reported significant improvement in her symptoms with no further abscess or painful breast lesions. On a follow-up visit, she complained of new-onset pain and discomfort in the left eye without photophobia or light sensitivity, or vision change for two weeks while on prednisone 20 mg daily, which ultimately was diagnosed as scleritis in the left eye by an ophthalmologist, which was treated with topical eye drops. To control long-term granulomatous mastitis secondary to GPA, she was started on oral methotrexate 15 mg weekly, and gradually prednisone was tapered to zero within three months. The follow-up visit here reflected no more issues of breast abscess, and recurrent mastitis was controlled with methotrexate alone.

## Discussion

GPA is a relatively uncommon autoimmune condition that can impact individuals of both genders, typically occurring between their third and seventh decades of life.

GPA is often initially misidentified as infections, malignancies, or inflammatory conditions, leading to frequent misdiagnoses. Breast involvement in GPA is rare. Multiple case reports have been published since its first description by Elsner and Harper in 1969 [3]. The presentation of breast involvement can range from a painless lump to a painful mass, accompanied by redness, hardening of the tissue, and occasionally ulceration [4]. In the majority of instances, breast lesions develop either simultaneously or sequentially as part of a multi-organ systemic disease, often accompanied by upper or lower airway symptoms. In less common scenarios, unilateral or bilateral breast lesions may emerge as the initial or sole symptom of the disease [5]. Goulabchand et al., in their literature review in 2020, found nine cases of GPA revealed by breast disease, initially restricted to the breast [6].

According to the discussion by Veerysamy et al., idiopathic granulomatous mastitis often manifests in premenopausal women who have given birth and can also develop during pregnancy The exact cause of this condition remains unclear, although hormonal imbalances, such as elevated prolactin levels observed in lactating breasts, are considered potential contributing factors [7].

Moreover, the occurrence of local trauma from mammary reduction has been observed to induce granulomatosis in a 25-year-old individual of Caucasian descent, presenting symptoms akin to Wegener's granulomatosis. In this context, skin lesions at the trauma site may have triggered local inflammation, cytokine production, and neutrophil activation. When combined with the presence of circulating c-ANCAs, this interplay could have contributed to the development of focal vasculitis at the surgical site, resembling the Koebner phenomenon. This aligns with the concept that ANCA-associated vasculitis follows a two-step process, requiring both the presence of ANCAs and an inflammatory trigger, typically infectious but in this instance, mechanical, to initiate neutrophil priming and activation [8].

There have been reported associations between GM and various autoimmune rheumatic diseases. Among these, the most prevalent is the granulomatous mastitis-erythema nodosum-arthritis syndrome (GMENA), along with granulomatosis with polyangiitis (Wegener's) and sarcoidosis [9].

Understanding the atypical presentation of GPA with breast involvement is essential for accurate diagnosis and timely intervention. Among the different disease-modifying anti-rheumatic drugs, methotrexate is considered a treatment option to treat relapsing or refractory idiopathic granulomatous mastitis to systemic steroids [10]. Given the rarity of this manifestation, it is crucial for healthcare professionals to consider GPA in the differential diagnosis when evaluating patients with recurrent mastitis. Further research and case studies are needed to enhance our understanding of the clinical characteristics and optimal management of GPA with breast involvement as granulomatous mastitis. In our case report, the patient did not have any other systemic features with no involvement of the upper or lower respiratory tract at the time of initial presentation and was repetitively misdiagnosed as infectious mastitis.

## Conclusions

Given the rarity of this manifestation, it is crucial for healthcare professionals to consider GPA in the differential diagnosis when evaluating patients with recurrent mastitis. Further research and case studies are needed to enhance our understanding of the clinical characteristics and optimal management of GPA with breast involvement as granulomatous mastitis. In our case report, the patient did not have any other systemic features with no involvement of the upper or lower respiratory tract at the time of initial presentation and was repetitively misdiagnosed as infectious mastitis.
